# Stress hormones promote growth of B16-F10 melanoma metastases: an interleukin 6- and glutathione-dependent mechanism

**DOI:** 10.1186/1479-5876-11-72

**Published:** 2013-03-22

**Authors:** Soraya L Valles, María Benlloch, María L Rodriguez, Salvador Mena, José A Pellicer, Miguel Asensi, Elena Obrador, José M Estrela

**Affiliations:** 1 Department of Physiology, University of Valencia, 15 Av. Blasco Ibañez, 46010, Valencia, Spain; 2 Faculty of Medicine, San Vicente Martir Catholic University, 2 Calle Quevedo, 46001, Valencia, Spain

**Keywords:** Metastases, Glutathione, Interleukin 6, Stress hormones, Apoptosis

## Abstract

**Background:**

Interleukin (IL)-6 (mainly of tumor origin) activates glutathione (GSH) release from hepatocytes and its interorgan transport to B16-F10 melanoma metastatic foci. We studied if this capacity to overproduce IL-6 is regulated by cancer cell-independent mechanisms.

**Methods:**

Murine B16-F10 melanoma cells were cultured, transfected with red fluorescent protein, injected i.v. into syngenic C57BL/6J mice to generate lung and liver metastases, and isolated from metastatic foci using high-performance cell sorting. Stress hormones and IL-6 levels were measured by ELISA, and CRH expression in the brain by *in situ* hybridization. DNA binding activity of NF-κB, CREB, AP-1, and NF-IL-6 was measured using specific transcription factor assay kits. IL-6 expression was measured by RT-PCR, and silencing was achieved by transfection of anti-IL-6 small interfering RNA. GSH was determined by HPLC. Cell death analysis was distinguished using fluorescence microscopy, TUNEL labeling, and flow cytometry techniques. Statistical analyses were performed using Student’s t test.

**Results:**

Plasma levels of stress-related hormones (adrenocorticotropin hormone, corticosterone, and noradrenaline) increased, following a circadian pattern and as compared to non-tumor controls, in mice bearing B16-F10 lung or liver metastases. Corticosterone and noradrenaline, at pathophysiological levels, increased expression and secretion of IL-6 in B16-F10 cells *in vitro*. Corticosterone- and noradrenaline-induced transcriptional up-regulation of IL-6 gene involves changes in the DNA binding activity of nuclear factor-κB, cAMP response element-binding protein, activator protein-1, and nuclear factor for IL-6. *In vivo* inoculation of B16-F10 cells transfected with anti-IL-6-siRNA, treatment with a glucocorticoid receptor blocker (RU-486) or with a β-adrenoceptor blocker (propranolol), increased hepatic GSH whereas decreased plasma IL-6 levels and metastatic growth. Corticosterone, but not NORA, also induced apoptotic cell death in metastatic cells with low GSH content.

**Conclusions:**

Our results describe an interorgan system where stress-related hormones, IL-6, and GSH coordinately regulate metastases growth.

## Background

Glutathione (GSH, γ-glutamyl-cysteinyl-glycine) is involved in cell protection against free radicals, and in many cellular functions being particularly relevant in cancer cells by regulating carcinogenic mechanisms; sensitivity against xenobiotics, ionizing radiation and some cytokines; DNA synthesis; cell proliferation; the protection against tumor microenvironment-related aggression, apoptosis evasion, colonizing ability, and multidrug and radiation resistance [1-3].

Recently, using the highly metastatic B16 melanoma F10 (B16-F10) cell line (a classical model with very high metastatic potential), we reported that interleukin 6 (IL-6) (mainly of tumor origin) facilitates GSH release from hepatocytes and its interorgan transport through the blood circulation to metastatic growing foci [4]. γ-Glutamyl transpeptidase (GGT) cleaves extracellular GSH, releasing γ-glutamyl amino acids and cysteinylglycine, which is further cleaved by membrane-bound dipeptidases into cysteine and glycine [1,5]. Free γ-glutamyl-amino acids, cysteine, and glycine entering the cell serve as GSH precursors [6]. In agreement with these facts, we found that tumor GGT activity and the intertissue flow of GSH, where the liver plays a key role, regulate GSH content of B16 melanoma cells and thereby their metastatic growth [7,8]. Nearly half of the GSH released by rat hepatocytes is transported across the sinusoidal membrane into the blood plasma for delivery to other tissues [9].

The role of IL-6 in cancer metastases is complex and may involve a) autocrine and paracrine mechanisms of IL-6 activity; b) direct growth stimulatory activity through activation of several signaling mechanisms; c) attraction of circulating immune and cancer cells to specific organs (e.g.lungs, brain, or liver) where IL-6 can be overexpressed; d) stimulation of neoangiogenesis and vascular remodeling; and e) promotion of inflammatory reactions and immune scape, thus contributing to an immune microenvironment that is favorable to tumor progression (e.g. [10-13]). In addition, anticancer treatments, such as the chemotherapeutic agents doxorubicin or paclitaxel and radiation therapy, can also facilitate IL-6 release by tumor cells [14]. Therefore, taking into account the pro-cancer roles of IL-6, it is not surprising that elevated serum levels of IL-6 and sIL-6R have been associated to chemoresistance with poor clinical outcome in different human cancers [15]. Many cancer cells, including e.g. prostate, breast, and colon cancer or melanoma, may produce large amounts of IL-6 and express the IL-6R/gp80 and gp130 receptor subunits, which allow them to respond to IL-6 stimulation even in an autocrine manner [13]. However, whether this capacity to overproduce IL-6 is constitutive in metastatic cells and/or regulated by cancer cell-independent mechanisms is unknown.

The nervous, endocrine, and immune systems interact trying to maintain physiological homeostasis under conditions that induce systemic cytokine production [16]. Stress has been suggested as a promoter of tumor growth and angiogenesis in different *in vivo* models [17]. The hypothalamus-pituitary-adrenal (HPA) axis, a main coordinator of the stress response, can be stimulated by cytokines (e.g. IL-1, IL-6, or αTNF) during the course of different immune, inflammatory, and neoplastic processes [18]. IL-6 is an essential corticotropin-releasing hormone (CRH)-independent stimulator of the pituitary-adrenal axis [19]. Activation of the HPA axis causes an increased secretion of adrenocorticotropin hormone (ACTH), which stimulates synthesis and release of glucocorticoids from the adrenal glands [20]. Glucocorticoids have been used widely in conjunction with other treatments for patients with cancer because (in addition to other potential benefits) they have proapoptotic properties in different cancer cell types; nevertheless glucocorticoids may also induce a resistant phenotype (still undefined) and, thereby, facilitate fast growth and metastases of different solid tumors [21]. Stress-related pathophysiological concentrations of cortisol have been shown to increase IL-6 production by human squamous cell carcinoma cells [22]. Besides, cancer associated-chronic stress can be associated in turn with a sympathetic system-induced increase in catecholamines production [23]. Moreover noradrenaline (NORA), at stress-related concentrations, has been shown to up-regulate VEGF, IL-, and IL-6 expression in different human melanoma cell lines [24].

Therefore it is plausible that glucocorticoids and/or catecholamines may influence IL-6 production by growing metastatic cells. The main objective of the present contribution was to explore the possibility that the IL-6/GSH interorgan cycle [4], as a metastases growth promoting activity, could be regulated by stress-related hormones under *in vivo* conditions.

## Materials and methods

### Culture of B16-F10 melanoma cells

Murine B16-F10 melanoma cells (from the ATCC, Rockville, MD) were cultured in serum-free Dulbecco's modified Eagle's medium (DMEM; Gibco, Grand Island, NY), pH 7.4, supplemented with 10 mM HEPES, 40 mM NaHCO_3_, 100 U/ml penicillin and 100 μg/ml streptomycin [8]. Cells were harvested by incubation for 5 min with 0.05% (w/v) trypsin (Sigma, St. Louis, MO) in PBS (10 mM sodium phosphate, 4 mM KCl, 137 mM NaCl), pH 7.4, containing 0.3 mM EDTA, followed by the addition of 10% calf serum to inactivate the trypsin. Cell numbers were determined using a Coulter Counter (Coulter Electronic Inc., Miami, FL). Cell integrity was assessed by trypan blue exclusion and leakage of lactate dehydrogenase activity [8].

### Animals

Syngenic male C57BL/6J mice (12 weeks old) from Charles River Laboratories (Barcelona, Spain) were fed ad libitum on a standard diet (Letica, Barcelona, Spain). Mice were kept on a 12-h light/12-h dark cycle with the room temperature maintained at 22°C. Procedures involving animals were in compliance with international laws and policies (EEC Directive 86/609, OJ L 358. 1, December 12, 1987; and NIH Guide for the Care and Use of Laboratory Animals, NIH Publ. No. 85-23, 1985). Experimental research on mice was performed with the approval of the ethics committee on animal research of the University of Valencia (Spain).

### Transfection of red fluorescent protein

The pDsRed-2 vector (Clontech Laboratories Inc., Palo Alto, CA) was used to engineer B16-F10 melanoma clones stably expressing red fluorescent protein (RFP). Cultured B16-F10 cells were transfected as previously described [4]. High-Performance Cell Sorting (DAKO, Copenhagen, Denmark) was used to select geneticin-resistant B16-F10 clones expressing the RFP (B16-F10-RFP) and showing high fluorescence emission. These cells were seeded in 96 wells plates, and their growth was followed by immune-fluorescence microscopy to select clones showing stable fluorescence emission.

### Experimental metastases

Hepatic or lung metastases were produced by i.v. injection (portal vein or tail vein, respectively) into anesthetized mice (Nembutal, 50 mg/kg i.p.) of 10^5^ viable B16-F10-RFP suspended in 0.2 ml DMEM. Mice were cervically dislocated 10 days after tumor cell inoculation. Livers and lungs were fixed with 4% formaldehyde in PBS (pH 7.4) for 24 hours at 4°C and then parafin-embedded. Metastases volume (mean % of organ volume occupied by metastases) was determined as earlier described [25].

### Isolation of B16-F10 melanoma cells from metastatic foci

Isolation of B16-F10 melanoma cells from metastatic foci was performed as previously described [4]. Briefly, tissues containing tumor cells were obtained by surgical means. Cell dispersion was carried out in minced tissue by trypsinization and collagenase digestion. Cells were washed three times in PBS and resuspended in 1 ml of ice-cold PBS, filtered through a 44-μm pore mesh and analyzed using a MoFlo High-Performance Cell Sorter (DAKO). Fluorescent B16-F10-RFP cells were separately gated for cell sorting and collected into individual tissue culture chambered slides (Nalge Nunc International Corp., Naperville, IL). Then the sorted tumor cells were harvested and plated in 25-cm^2^ polystyrene flasks (Falcon Labware).

### Measurement of adrenocorticotropin hormone, corticosterone, and norepinephrine levels

Plasma levels of ACTH (Calbiotech, Inc., Spring Valley, CA), corticosterone (Kamiyama Biomedical Co., Seattle, WA), and NORA (IBL, Hamburg, Germany) were quantified by ELISA according to the instructions of the suppliers.

### CRH expression in the brain (*in situ* hybridization)

Sections of 10 μm of the paraventricular nucleus (PVN) were cut according to a mouse brain atlas (Allen Insttute for brain science, http://www.brain-map.org) on a cryostat, mounted on polysine microscope slides (Menzel-Gläzer, Braunschweig, Germany), and stored at -80°C for 24 h. Then sections were fixed in 4% paraformaldehyde, further permeabilized by proteinase K treatment, acetylated twice with 0.25% acetic anhydride in 0.1 M triethanolamine, and dehydrated in a graded ethanol series.

Hybridization, carried out as described before [26], was performed using specific 48-mer, ^35^S-labeled oligonucleotide probes for murine CRH mRNA (5^′^-GGC CCG CGG CGC TCC AGA GAC GGA TCC CCT GCT CAG CAG GGC CCT GCA-3^′^) [27]. Hybridized slices were exposed to BioMax MR film (Kodak, Rochester, NY). The mRNA expression of CRH in the PVN was quantified as gray density minus background in digitized images using the NIH ImageJ 1.6 program (http://rsb.info.nih.gov/ij). Bilateral measures were taken from two to four PVN sections for each mouse, which were pooled to provide individual means per mouse. For tissue background, the optical density of a nonhybridized region outside the PVN was measured.

### Measurement of IL-6 levels

Blood samples were centrifuged at 14,000 rpm for 10 min at 4°C to separate the serum. Concentration of IL-6 in the serum was determined using commercially available mouse cytokine ELISA kits from Innovative Research (Novi, MI).

### DNA binding activity of NF-κB, CREB, AP-1, and NF-IL-6

Nuclear extracts were prepared with a nuclear extraction kit (Millipore, Billerica, MA). The DNA binding activity of nuclear factor-κB (NF-κB, p65/p50) and activator protein-1 (AP-1, c-Jun/c-Fos) in nuclear extract was determined by the NF-κB or AP-1 EZ-TFA transcription factor assay kits (Millipore) according to the manufacturer’s protocols; whereas the DNA binding activity of cAMP response element-binding protein (CREB) and nuclear factor for IL-6 expression (NF-IL-6) were determined by specific ELISA-based TransAm™ (Active Motif North America, Carlsbad, CA) assay kits following manufacturer’s procedures.

### Transfection of anti-IL-6 small interfering RNA

IL-6 silencing was performed as previously described in detail [4]. The PSilencer 3.1-H1 linear vector from Ambion Inc. (Austin, TX) was used to obtain long term gene silencing. The siRNA molecules targeting IL-6 mRNA were purchased from Ambion. The RNA duplex against IL-6 had the sequence 5^′^-GGA CAU GAC AAC UCA UCU CTT-3^′^ (sense) and 5^′^-GAG AUG AGU UGU CAU GUC CTG-3^′^ (antisense). The negative control vector that expresses a hairpin siRNA with limited homology to any known sequences in mice was provided by the vector kit (Ambion). Stably transfected clones were selected in medium containing 0.5 mg/ml Geneticin (Invitrogen). Established clones were grown in medium supplemented with 10% FCS and 0.5 mg/ml Geneticin. Silencing was confirmed by immunoblotting.

### GSH determination

GSH was determined, following procedures previously described [28], by liquid chromatography-mass spectrometry using a Quattro micro triple-quadrupole mass spectrometer (Micromass, Manchester, UK) equipped with a Shimadzu LC-10ADVP pump and SCL-10AVP controller system with an SIL-10ADVP autoinjector (Shimadzu Corporation, Kyoto, Japan). Cell processing was performed according to published methodology, where rapid N-ethylmaleimide derivatization was used to prevent GSH auto-oxidation [29].

### Cell death and cell cycle analysis

Apoptotic and necrotic cell death were distinguished by using fluorescence microscopy. For this purpose, isolated cells were incubated with Hoescht 33342 (10 mM; which stains all nuclei) and propidium iodide (10 mM; which stains nuclei of cells with a disrupted plasma membrane), for 3 min, and analyzed using a Diaphot 300 fluorescence microscope (Nikon, Tokyo, Japan) with excitation at 360 nm. Nuclei of viable, necrotic, and apoptotic cells were observed as blue round nuclei, pink round nuclei, and fragmented blue or pink nuclei, respectively. About 1,000 cells were counted each time. DNA strand breaks in apoptotic cells were assayed by using a direct TUNEL labeling assay (Boehringer, Mannheim, Germany) and fluorescence microscopy following manufacturer’s methodology. Quantitative determination of mitochondrial membrane potential, measurement of H_2_O_2_, flow cytometry determination of O_2_^.-^ generation, and measurements of cytochrome c release and caspase 3 activity, were performed as previously described [30]. Cell-cycle phase distribution was determined by analytical DNA flow cytometry as previously described [25].

### Compartmentation of B16-F10 cells

Cultured cells were harvested (see above), washed twice in DMEM, and resuspended in ice-cold Krebs-Henseleit bicarbonate medium (pH 7.4). Rapid separation of cytosolic and mitochondrial compartments, and calculation of mitochondrial volume, were performed as previously described [31].

### Cellular electroporation

Transient plasma membrane permeabilization was obtained using an electroporation unit for eukaryotic cells (BioRad, Hercules, CA). The field strength applied to each sample was of 1.0 kV/cm with a time constant of 50 ms.

### RT-PCR and detection of mRNA

Total RNA was isolated using the TRIzol kit from Invitrogen and following the manufacturer’s instructions. cDNA was obtained using a random hexamer primer and a MultiScribe Reverse Transcriptase kit, as described by the manufacturer (TaqMan RT Reagents, Applied Biosystems, Foster City, CA). APCR master mix and AmpliTaq Gold DNA polymerase (Applied Biosystems) were then added containing the specific primers (Sigma-Genosys) previously reported [4] for *IL-6* and glyceraldehyde-3P-dehydrogenase (*GAPDH*). Real-time quantitation of the mRNA relative to GAPDH was performed with a SYBR Green I assay, and a iCycler detection system (Biorad, Hercules, CA). Target cDNA was amplified as follows: 10 min at 95°C, then 40 cycles of amplification (denaturation at 95°C for 30 sec and annealing and extension at 60°C for 1 min per cycle). The increase in fluorescence was measured in real time during the extension step. The threshold cycle (C_T_) was determined, and then the relative gene expression was expressed as: fold change= 2^–Δ(Δ^ ^C^ _T_ ^)^ , where Δ C_T_ = C_T_ target – C_T_ GAPDH, and Δ (Δ C_T_) = Δ C_T_ treated - Δ C_T_ control.

### Expression of results and statistical analyses

Data are presented as the means + S.D. for the indicated number of different experiments. Statistical analyses were performed using Student’s t test, and P values < 0.05 were considered significant.

## Results

### Stress hormones in metastatic tumor-bearing mice

**Figure 1:**
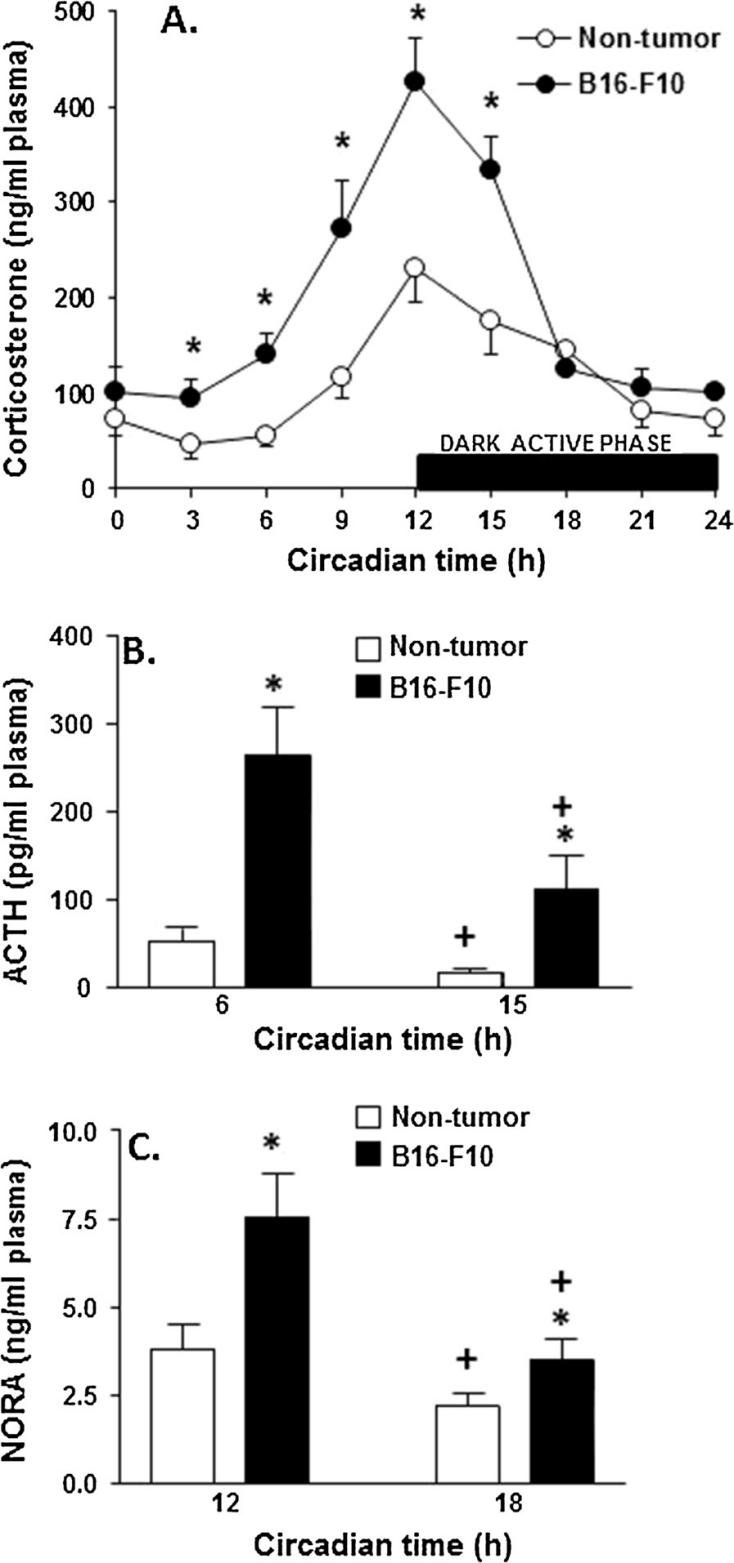
**Corticosterone, ACTH, and noradrenaline levels in plasma of non-tumor- and B16-F10-bearing (lung metastases) mice.** Corticosterone, ACTH, and noradrenaline levels were measured as indicated under Materials and methods. Blood was collected from the tail vein during the 24-h period. Data are mean values + S.D. (*error bars*) of 7–8 different animals. *P< 0.05 comparing B16-F10 (7 days after tumor inoculation)-bearing mice versus non-tumor-bearing mice; +P< 0.05 comparing 15 h versus 6 h.

Stress-relative responses in rodents under stressful conditions can be evaluated by measuring plasma levels of corticosterone and NORA (main circulating glucocorticoid and catecholamine, respectively) [32,33]. As shown in Figure 1. A corticosterone levels in plasma peak at 12 h, right before the begin of the dark active phase in mice. However corticosterone levels were significantly higher in B16-F10 (lung metastases)-bearing mice than in control non-tumor-bearing mice (Figure 1A). In agreement with this finding plasmatic ACTH levels were also higher in metastases-bearing mice than in controls, and also followed a circadian pattern (ACTH was higher before corticosterone levels peaked, and lower during the dark active phase) (Figure 1B). Besides NORA levels in plasma were also higher in metastases-bearing mice than in controls (Figure 1C). Similar results were found in mice bearing B16-F10 metastases growing in the liver (not shown), thus suggesting a general mechanism not dependent on the site of metastases growth.

### Corticosterone and noradrenaline stimulate IL-6 expression and secretion in metastatic cells

Our next step was to investigate if corticosterone and/or NORA, at pathophysiologically relevant concentrations, could influence IL-6 expression and/or secretion in metastatic cells. For this purpose B16-F10 cells were cultured in the presence of corticosterone and/or NORA, which were incubated at mean peak plasmatic values (approx. 350 ng corticosterone/ml and/or 5.5 ng NORA/ml in mice bearing B16-F10 metastases versus 150 ng corticosterone/ml and/or 3.0 ng NORA/ml in control non-tumor-bearing mice) for a period of 6 h (see Figure 1). As a double control, B16-F10-RFP cells isolated from lung metastases were also assayed. A shown in Table 1, both corticosterone and NORA significantly increase IL-6 expression and secretion in B16-F10 cells. Although, when both hormones were added together, IL-6 expression and secretion values were not significantly different from those found using corticosterone alone (Table 1). Moreover, B16-F10-RFP cells (isolated from lung metastatic growing foci) showed higher IL-6 expression and secretion as compared to control B16-F10 cells, which is not surprising since these cells have been exposed to higher corticosterone and NORA levels under *in vivo* conditions. In agreement with this idea, *in vitro* exposure to B16-F10-RFP cells to corticosterone and/or NORA did not up-regulate IL-6 expression and/or secretion as compared to B16-F10-RFP controls (Table 1).

**Table 1:** **Effect of corticosterone and NORA on IL-6 expression and secretion by B16-F10 and B16-F10-RFP cells *in vitro***

**Tumor cells**	**Additions**	**Expression (-fold change)**	**Secretion (pg/10**^**6 **^**cells x 24 h)**
B16-F10	None	1.0 ± 0.1	833 ± 170
	Corticosterone	2.5 ± 0.4^*^	1654 ± 266^*^
	NORA	1.7 ± 0.2^*^	1257 ± 214^*^
	Corticosterone+NORA	2.4 ± 0.3^*^	1712 ± 247^*^
B16-F10-RFP	None	3.0 ± 0.3^*+^	1966 ± 331^*+^
	Corticosterone	3.3 ± 0.4^*+^	2184 ± 287^*+^
	NORA	3.0 ± 0.3^*+^	1763 ± 294^*+^
	Corticosterone+NORA	3.6 ± 0.5^*+^	2477 ± 315^*+^

IL-6 expression and release to the extracellular medium were measured (48 and 72 h after seeding, respectively) as indicated under Materials and methods. Corticosterone and/or NORA were incubated (at the concentrations indicated in the text) 24 h after seeding and were present in the incubation medium for 6 h, then the medium was renewed. Data are mean values + S.D. (n= 5-6) calculated for the indicated period. *P< 0.01 comparing both cell types and all conditions versus control B16-F10 cells; +P< 0.01 comparing all additions under B16-F10-FRP cells versus their equivalents under B16-F10 cells.

Human and murine melanoma cells express high-affinity glucocorticoid receptors (GCRs) [34]; and the presence of adrenoceptors (ARs) has been also detected in different melanoma cells [24]. Moreover, the presence of GCRs [35] and β ARs [36] in B16 melanoma cells has been reported. Thus, we investigated if corticosterone- and NORA- induced up-regulation of metastatic cell IL-6 production is a mechanism specifically bound to GCRs and/or ARs. For this purpose we used mifepristone (RU-486) to block GCRs [37] and propranolol to block β ARs [38]. Addition of RU-486 (50 μM) or propranolol (50 μM) to cultured B16-F10 cells (2 h before hormones addition) completely abolished the corticosterone- or NORA-induced increase in IL-6 secretion displayed in Table 1 (data not shown). The ARs-mediated effect was specific to β ARs since the α-adrenergic antagonist prazosin (5 μM) had no effect on the ability of corticosterone and/or NORA to induce IL-6 expression (not shown). Therefore corticosterone and NORA-induced up-regulation of IL-6 in metastatic cells indeed appears to involve interaction of these hormones with their specific receptors.

### Transcriptional regulation of the IL-6 gene by corticosterone and noradrenaline

**Figure 2:**
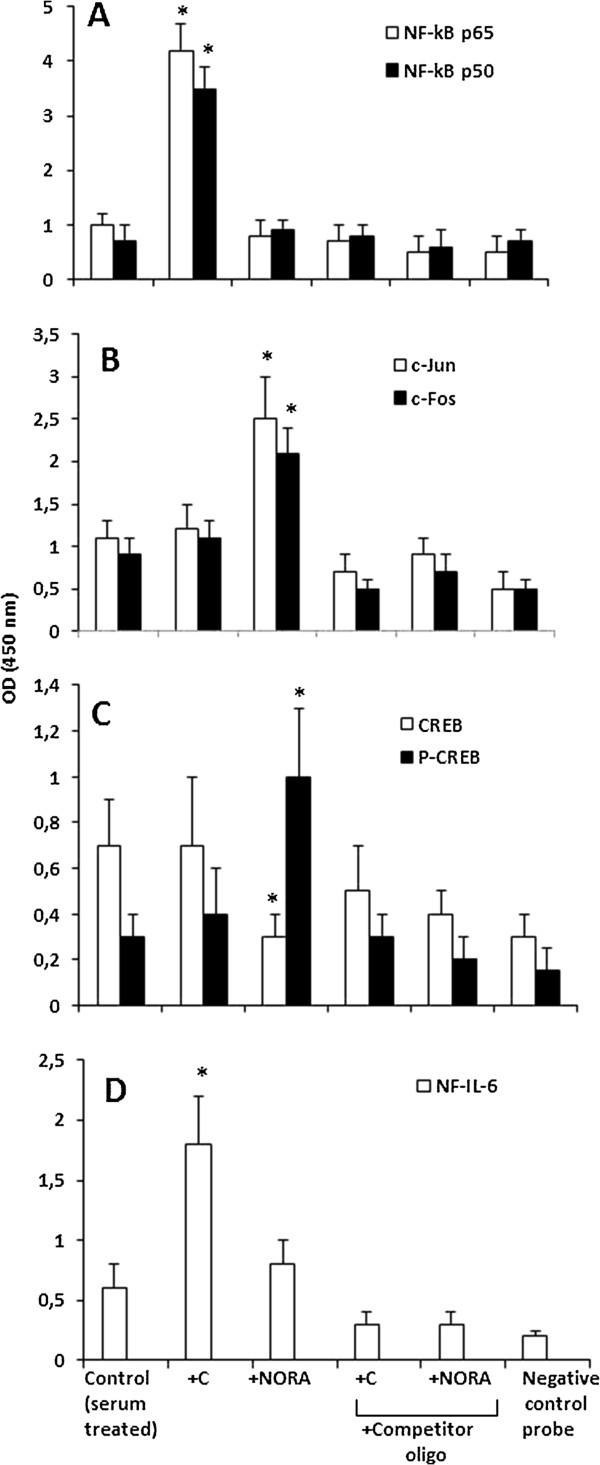
**NF-κB, CREB, AP-1, and NF-IL-6 DNA binding activity in nuclear extracts of B16-F10 melanoma cells treated with corticosterone or NORA.** Corticosterone (**C**) and NORA were incubated (at the concentrations indicated in Table 1) 18 h after seeding and were present in the incubation medium for 6 h. Nuclear extracts were then obtained as described under Materials and methods. Results are means + S.D. (*error bars*) of 4-5 independent experiments. The significance test refers to the comparison between each experimental condition versus controls (serum treated) (*P< 0.01).

Previous work by several groups showed that NF-κB, NF-IL-6, AP-1, CREB, interferon-regulatory factor-1, and specificity protein 1 can interact with the IL-6 promoter to initiate mRNA synthesis [13]. GCRs coordinate with NF-κB to regulate expression of different pro-inflammatory cytokines, including IL-6 [39]. Whereas β-adrenergic stimulation, via the cAMP-protein kinase A signaling pathway, leads to activation of AP-1 [40]. Therefore, we investigated the effect of these hormones on activation of NF-κB, CREB, AP-1, and NF-IL-6, which correspond to the four major transcriptional regulatory sites present in the IL-6 promoter region of the IL-6 gene [13]. As shown in Figure 2, corticosterone increases DNA binding activity of NF-κB (p65 and p50) in cultured B16-F10 cells (Figure 2A), whereas NORA increases DNA binding activity of phosphorylated CREB (P-CREB, which in addition can mediate β-adrenergic stimulation of c-Fos via protein kinase A [41] (Figure 2C) and the AP-1 complex (c-Jun and c-Fos) (Figure 2B). DNA binding activity of NF-IL-6 was significantly increased by corticosterone but not by NORA (Figure 2D), which is interesting because single binding sites for NF-IL6 and NF-kappa B are present in the promoter of the IL 6 gene [42]. In fact NF-IL6 and NF-kappa B synergistically activate transcription of IL-6 and other cytokines [42]. Therefore interaction of corticosterone and NORA with their receptors is linked, via intracellular signaling cascades, with the molecular mechanism promoting IL-6 expression.

Cell cycle distribution (approximate percentage of cells in G0/G1, S, and G2/M phases, n = 5) was 48.0 ± 5.2, 29.4 ± 3.4, and 23.6 ± 2.4 in controls growing exponentially (24 h after seeding, as the controls in Figure 2); and 66.0± 4.2, 18.2 ± 1.8, and 15.8 ± 2.6 in cells close to quiescence (66 h after seeding). However results reported in Figure 2 (Effect of corticosterone and NORA on activation of NF-κB, CREB, AP-1, and NF-IL-6) for cells cultured × 24 were not significantly different from those found in cells cultured × 66 h. Thus indicating that, in the standard cultured conditions used in our experiments, GCRs- and ARs-dependent signaling mechanisms (and likely expression of these receptors) are not affected by the cell cycle distribution along the culture time.

### Tumor-derived IL-6 facilitates activation of the pituitary-adrenal axis

**Figure 3:**
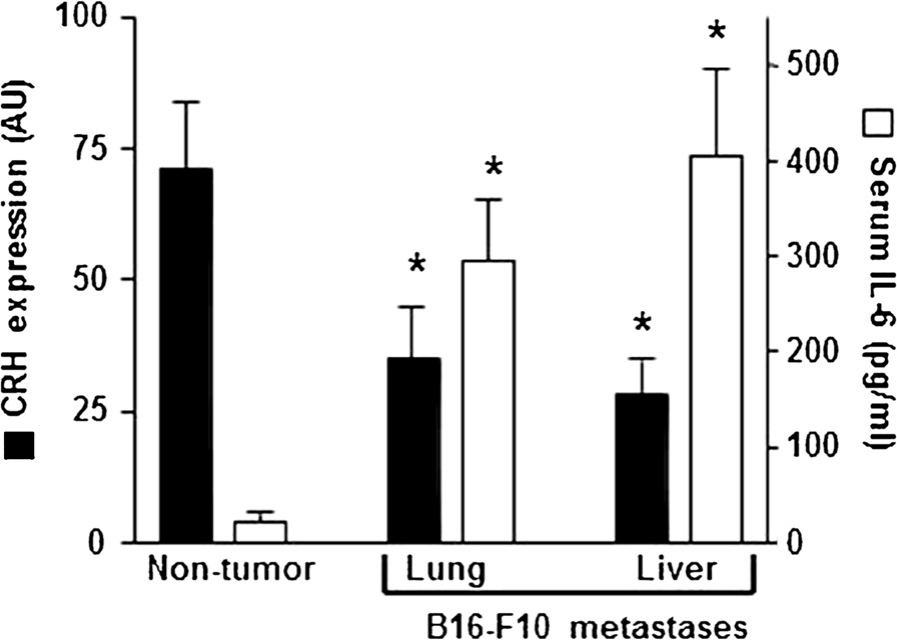
**CRH expression and serum IL-6 levels in non-tumor- and B16-F10 (metastases)-bearing mice.** Measurements were performed in non-tumor-bearing and in tumor-bearing mice (7 days after inoculation). mRNA expression of CRH in the hypothalamic PVN was evaluated as described under Materials and methods. CRH expression data (optical density arbitrary units, AU), measured at 0 h (circadian time, see Figure 1), are expressed as mean values + S.D. (error bars) of 6–7 different animals. For IL-6 levels determination blood was collected from the tail vein every 6 h during the 24-h period of the indicated day, and data are mean values of the peak serum cytokine concentrations + S.D. (*error bars*) (pg/ml) measured in 7–8 different animals. *P< 0.01 comparing B16-F10-bearing mice versus non-tumor-bearing mice.

The major neuroendocrine response mediating stress adaptation is activation of the HPA axis, with stimulation of CRH and vasopressin, leading to pituitary ACTH secretion and increases in glucocorticoid secretion from the adrenal cortex [43]. Tumor derived cytokines have been suggested to activate the HPA axis [44,45]. Interestingly IL-6 in particular appears essential for activation of the HPA axis during immunological challenge in the absence of hypothalamic input from CRH [19]. Moreover it has been reported that suppressor of cytokine signaling-3, stimulated by IL-6 and cAMP, is involved in the negative regulation of CRF gene expression [46]. Thus we investigated if, in mice bearing B16-F10 melanoma metastases, increased circulating IL-6 did affect CRH production. As shown in Figure 3, CRH expression in the hypothalamic PVN was significantly lower in metastatic B16-F10-bearing mice than in non-tumor-bearing controls, whereas serum IL-6 increased. Moreover, CRH expression was similar in mice bearing lung or liver metastases (Figure 3). Thus suggesting again a systemic mechanism which does not depend on the site of metastatic growth. To investigate if tumor-derived IL-6 is directly linked to changes in CRH expression under *in vivo* conditions, we inoculated intravenously control B16-F10 cells and B16-F10 cells transfected with siRNA specific for IL-6 (B16-F10/IL-6-siRNA). As shown in Table 2, down regulation of tumor IL-6 expression in mice bearing B16-F10/IL-6-siRNA metastases associated with a decrease in circulating IL-6 levels. Besides CRH expression increased in B16-F10/IL-6-siRNA-bearing metastases as compared to B16-F10 controls, whereas ACTH levels were similar in both cases (Table 2). Thus indicating, as previously suggested [19], that IL-6 may act in metastatic tumor-bearing hosts as a CRH-independent pituitary stimulator.

**Table 2:** Effect of siRNA-induced tumor IL-6 silencing on hypothalamic CRH expression and circulating levels of IL-6 and ACTH in B16-F10 tumor-bearing mice

	**Lung metastases**	
	**B16-F10**	**B16-F10/IL-6-siRNA**
Tumor IL-6 expression (-fold change)	1.0 ± 0.2	0.15 ± 0.05^*^
Serum IL-6 (pg/ml)	347 ± 75	132 ± 34^*^
Hypothalamic CRH expression (AU)	40 ± 12	85 ± 16^*^
Plasma ACTH (pg/ml)	240 ± 50	255 ± 63

Gene transfections were performed as explained under Materials and methods. CRH expression data are expressed as optical density arbitrary units (AU). All measurements were performed 7 days after tumor cell inoculation. The data show mean values ± S.D. for 5–6 different experiments. *P< 0.01 comparing B16-F10/IL-6-siRNA-bearing mice versus control B16-F10-bearing mice.

It is also plausible that CRH expression could be influenced by IL-6-dependent factors (missed in B16-F10 with IL-6 expression silencing). Nevertheless, as shown in Table 2, tumor IL-6 silencing increases (x 2-fold) hypothalamic CRH expression in B16-F10 tumor-bearing mice. Besides tumor IL-6 silencing associates with a decrease in serum IL-6 (approx. 70% less in mice bearing lung or liver metastases, Table 3). Thus, without ruling out the possibility mentioned above, down-regulation of tumor IL-6 appears a main factor influencing hypothalamic CRH expression. A fact anticipated by the work of Bethin et al. [19] and confirmed by our studies.

**Table 3:** ***In vivo *effect of RU-486, propranolol, and siRNA-induced down regulation of tumor IL-6 expression, on circulating levels of IL-6, corticosterone and NORA, hepatic GSH and metastases growth**

	**Treatment**							
	**Physiological saline**		**RU-486**		**Propranolol**		**IL-6-siRNA**	
**Metastases**	**Lung**	**Liver**	**Lung**	**Liver**	**Lung**	**Liver**	**Lung**	**Liver**
Tumor IL-6 expression (-fold change)	1.0 ± 0.1	1.0 ± 0.2	0.4 ± 0.1^**^	0.3 ± 0.15^**^	0.6 ± 0.2^**^	0.7 ± 0.15^*^	0.15 ± 0.05^**^	0.2 ± 0.1^**^
Serum IL-6 (pg/ml)	355 ± 81	432 ± 69	123 ± 36^**^	154 ± 49^**^	206 ± 77^*^	255 ± 60^**^	107 ± 40^**^	124 ± 33^**^
Liver GSH (μmol/g of tissue)	4.5 ± 05	3.8 ± 0.4	6.8 ± 0.6^**^	5.9 ± 0.5^**^	6.2 ± 0.5^**^	5.5 ± 0.5^**^	7.3 ± 0.6^**^	7.1 ± 0.5^**^
Plasma NORA (ng/ml)	406 ± 65	447 ± 72	337 ± 59	412 ± 77	426 ± 68	394 ± 59	433 ± 67	360 ± 45^*^
Plasma NORA (ng/ml)	7.9 ± 1.5	8.7 ± 1.4	7.3 ± 1.2	8.1 ± 1.6	7.5 ± 1.1	8.5 ± 1.7	8.2 ± 1.3	9.2 ± 1.6
Metastases volume (%)	6.4 ± 09	8.5 ± 1.2	4.0 ± 0.6^**^	5.0 ± 0.7^**^	4.9 ± 0.5^**^	5.8 ± 0.9^**^	3.0 ± 0.7^**^	3.7 ± 0.8^**^

RU-486 (20 mg/kg body wt.) [47] or propranolol (10 mg/kg body wt.) [48] were administered i.p. with daily frequency, starting 24 h after tumor cell inoculation. All measurements (see under Material and methods) were performed 7 days after tumor cell inoculation. Plasma levels of corticosterone and NORA were measured at 12 h (circadian time, see Figure 1). Metastases volume is indicated as the mean percentage of organ volume occupied by metastases. The data show mean values + S.D. for 9–10 different experiments. **P< 0.01, *P< 0.05 comparing each condition versus physiological saline-treated controls.

### Treatment with glucocorticoid receptor or β adrenoceptor blockers inhibits metastatic growth

Whether a decrease in circulating IL-6 levels was linked to metastatic activity, and whether this mechanism was regulated by corticosterone and/or NORA, was our next step forward. To address these key questions we compared physiological saline-treated B16-F10 metastases (lung or liver)-bearing controls with metastases-bearing mice treated with the GCR blocker (RU-486) or the β AR blocker (propranolol), and with mice inoculated with B16-F10/IL-6-siRNA cells. As shown in Table 3, tumor IL-6 expression and circulating IL-6 levels decreased in all groups as compared to controls, whereas hepatic GSH levels increased. However, plasma levels of corticosterone and NORA were significantly similar in all groups (Table 3). The decrease in circulating IL-6 levels found in RU-486- or propranolol-treated mice, or in mice inoculated with B16-F10/IL-6-siRNA cells, associated with lower metastases growth either in lung or liver (Table 3). However, metastases volume in mice inoculated with B16-F10/IL-6-siRNA cells was smaller (P< 0.05) than that found in RU-486- or propranolol-treated mice (Table 3). Thus suggesting that, perhaps, pathophysiological levels of corticosterone and/or NORA have some anti-cancer effects which are absent if GCRs and/or β ARs are blocked.

Glucocorticoids (such as dexamethasone) are widely used in cancer therapy and may have cell type-specific pro- or antiapoptotic effects, although when applied at high therapeutic doses their anti-tumor effects prevail [34]. Nevertheless there are very limited data regarding possible direct effects of stress hormones, at *in vivo* pathophysiological levels, on cancer cell proliferation [49]. Some early findings in leukemia research suggested a possible link between GSH and glucocorticoids effects in cancer cells [50]. Maung et al. [51] found in their study of newly diagnosed leukemia patients a positive correlation between GSH levels and prednisolone resistance. Later Anderer et al. [52] reported the implication of polymorphisms in the GSH-S-transferase genes for glucocorticoid sensitivity in childhood acute leukemia. Besides, it has been shown that GSH levels in metastatic cells can regulate growth and death mechanisms [2,3]. However, whether GSH levels in metastatic cells influence stress hormones effects is unknown. Thus, in the next step, we investigated if corticosterone and/or NORA regulate growth and/or death mechanisms in B16-F10 cells, and if these effects are GSH dependent.

### Corticosterone induces cell death in metastatic cells with low GSH content

We evaluated the effect of corticosterone and NORA (at pathophysiological concentrations) on cell growth and viability using B16-F10 cell subsets with different GSH content. B16-F10 cells cultured to low density (12 h after seeding) show a high GSH content (40 + 5 nmol/10^6^ cells, n=7); whereas these cells, when incubated in the presence of 1 mM BSO (L-buthionine (SR)-sulphoximine, the non-toxic and selective GSH synthesis inhibitor [1]), show a low GSH content (14 + 3 nmol/10^6^ cells, n=7) under the same culture conditions. As shown in Table 4, corticosterone (but not NORA) decreased growth and viability of B16-F10 cells with low GSH content but not of those with high GSH content.

**Table 4:** Effect of corticosterone and NORA on growth and viability of B16-F10 cell subsets with different GSH contents

		**Culture time**					
		**12 h**			**48 h**		
**B16-F10 cells**	**Additions**	**GSH (nmol/10**^**6 **^**cells)**	**Cell number (10**^**6 **^**cells)**		**GSH (nmol/10**^**6 **^**cells)**	**Cell number (10**^**6 **^**cells)**	
			**Viable**	**Dead**		**Viable**	**Dead**
High GSH content							
	No addition	40 ± 5	2.01 ± 0.26	0.02 ± 0.01	46± 7	5.24 ± 0.36^*^	0.15 ± 0.04^*^
	+ Corticosterone					5.06 ± 0.17	0.18 ± 0.03
	+NORA					5.57 ± 0.43	0.14 ± 0.03
	+Corticosterone+NORA					4.82 ± 0.30	0.20 ± 0.05
Low GSH content							
	No addition	14 ± 3^+^	1.93 ± 0.15	0.05 ± 0.01^+^	8 ± 2	2.24 ± 0.45^+^	0.15 ± 0.03^*^
	+ Corticosterone					0.86 ± 0.15^+^	0.76 ± 0.14^+^
	+NORA					2.07 ± 0.26^+^	0.13 ± 0.05
	+Corticosterone+NORA					0.75 ± 0.18^+^	0.80 ± 0.17^+^

Cells were cultured as described under Materials and methods. To allow maintenance of a high intracellular GSH content GSH ester (1 mM) was added 12 h after seeding. To maintain a low intracellular GSH content BSO (200 μM) was added 2 h after seeding. Corticosterone and/or NORA were incubated (at the concentrations indicated in Table 1) 18 h after seeding and were present in the incubation medium for 6 h, then the medium was renewed and GSH ester and BSO were added again. Data are mean values + S.D. (n= 7) calculated for the indicated period. *P< 0.01 comparing 42h of culture time versus 12 h; +P< 0.01 comparing low versus high GSH content.

**Figure 4:**
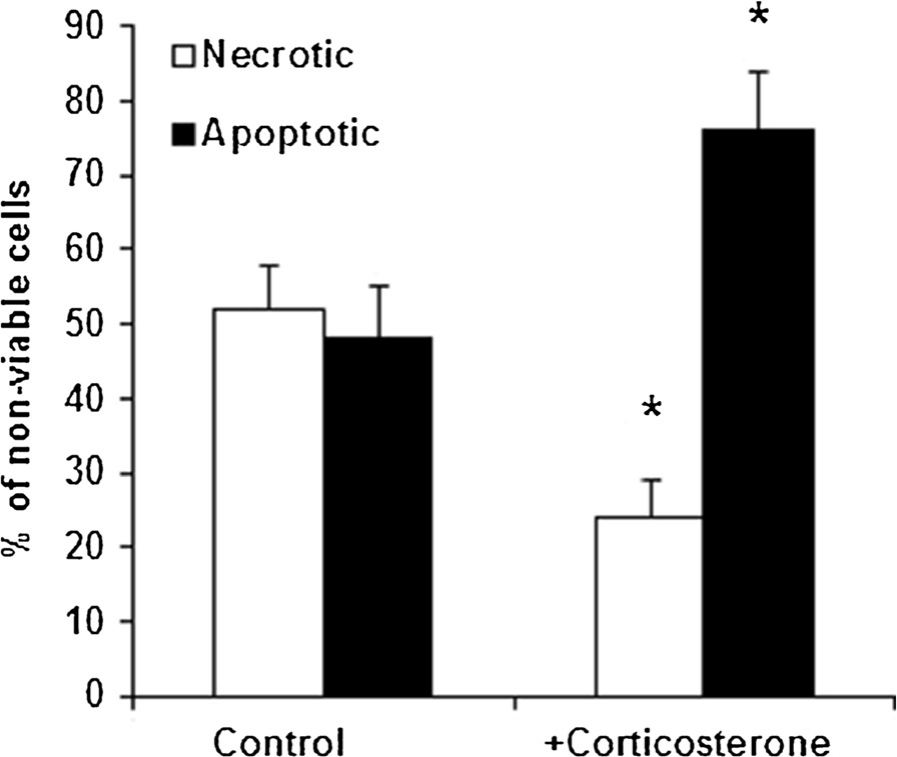
**Type of death induced by corticosterone in B16-F10 melanoma cells with low GSH content.** The experimental procedure was that indicated in Table 4. Cell death was analyzed using fluorescence microscopy as described under Materials and methods. Cell death analysis was performed every 2 h during the 24 h period after corticosterone removal from the cultured medium. Non-viable cells includes all cells marked as necrotic or apoptotic along the experiment. Data are mean values + S.D. (*error bars*) (n= 5-6) calculated for the indicated period. *P< 0.01 comparing cells treated with corticosterone versus control B16-F10 cells.

Corticosterone-induced cell death in B16-F10 cells with low GSH content was further analyzed. As shown in Figure 4, as a consequence of exposure to corticosterone, most dying cells displayed apoptotic features and only a small percentage was identified as necrotic. The percentage of apoptotic cells obtained by using Hoescht 33342 and propidium iodide or the TUNEL technique (see under Materials and methods) was similar (not shown). Molecular activation of apoptosis in B16-F10 cells with low GSH content was confirmed as shown in Table 5, where corticosterone-induced reactive oxygen species (ROS) generation (being mitochondria their principal source in cells) associates with mitochondrial GSH (mtGSH) and ATP depletion, a decrease in mitochondrial membrane potential, and an increase in cytosolic cytochrome c level and caspase 3 activity.

**Table 5:** ROS generation and the molecular activation of apoptosis upon corticosterone administration to B16-F10 cells with low GSH content

**Parameters**	**Controls**	**+ Corticosterone**	**+Corticosterone**
			**+GSH ester**
H_2_O_2_ (nmol/10^6^ cells)	0.45 ± 0.09	1.74 ± 0.35*	0.73± 0.20*^+^
O_2_^.-^ (ΔFL1)	2.10 ± 0.39	4.33 ± 0.41*	2.65 ± 0.27^+^
mtGSH (nmol/10^6^ cells)	3.2 ± 0.5	1.6 ± 0.4*	6.4 ± 1.0*^+^
MMP (TPM accumulation ration ratio, %)	95 ± 4	52 ± 9	90 ± 7^+^
mtATP (mM)	1.07 ± 0.24	0.66 ± 0.23	0.95 ± 0.18
Cytosolic cytochrome C (% of control)	100 ± 11	165 ± 27	112 ± 15^+^
Caspase 3 (pmol/10^6^ cells x min)	1.36 ± 0.21	3.15 ± 0.77	1.79 ± 0.26^+^

Corticosterone was added as indicated in the caption to Table 1. GSH ester (1 mM) was added 6 h before corticosterone addition. BSO was added to all cells as indicated in the caption to Table 4. Measurements were performed 12 h after corticosterone removal. Data are means + S.D. of 5 or 6 different experiments. *P< 0.01 comparing all conditions versus untreated controls; +P< 0.01 comparing cells treated with corticosterone + GSH ester versus those treated with corticosterone alone.

Depletion of mtGSH (which cannot be synthesized by mitochondria and must be transported from the cytosol) may facilitate mitochondrial membrane permeabilization, permeability transition pore opening, and the release of apoptosis-inducing molecular signals [30]. Indeed when GSH levels were increased, after loading the cells with GSH ester (which readily enters the cell and delivers free GSH, [30]), mtGSH increased and the mitochondria dysfunction-dependent/corticosterone-induced molecular activation of cell death was prevented (Table 4). To prove that mtGSH is directly involved (and because there are no specific inhibitor of the GSH transport into mitochondria) we used cell electroporation in the presence of 10 mM L-glutamate (a competitive inhibitor of the mtGSH transport). In the presence of BSO, GSH ester, and L-glutamate, cytosolic GSH levels in B16-F10 cells increased up to 42 + 6 nmol/10^6^ cells (n=6) (see control values in Table 4); whereas when corticosterone was added mtGSH remained low (2.0 + 0.4 nmol/10^6^ cells) (n=6) (see control values in Table 5) and the molecular activation of apoptosis (as in Table 5) was not prevented (data not shown). Thus indicating that cellular GSH, and mtGSH in particular, regulate the mechanism of corticosterone-induced metastatic cell death.

## Discussion

### Neuroendocrine mediators, IL-6, and metastases growth

In the present work we have observed that stress-related hormones (corticosterone and NORA) promote overexpression of IL-6 in metastatic B16-F10 cells (Table 1). Plasmatic levels of these hormones (including pituitary ACTH) follow a physiological circadian pattern, but were higher in metastases-bearing mice (Figure 1). To date the majority of neuroendocrinological research dealing with stress and accelerated tumor growth has focused on suppressed immune response to malignant tissue [53]. However recent molecular and animal studies have begun to identify specific signaling pathways suggesting an impact of neuroendocrine mediators on tumor growth and metastasis [49]. Nevertheless, as shown by different studies (mainly *in vitro*), the effects of stress-related hormones on tumor cell proliferation can be either stimulatory or inhibitory [53]. This apparent paradox may depend, as it has been suggested, on (yet undefined) differences between cancer cells [53].

### Corticosterone, GSH, and metastatic cell viability

Here we show that GSH levels in metastatic cells, which are regulated by the IL-6/GSH interorgan cycling activity (where the liver provides GSH to metastases) [4], directly influence the effect of corticosterone on tumor cell viability (Table 4). Pathophysiologically relevant levels of corticosterone or NORA increase IL-6 secretion by B16-F10 cells (Table 1), which subsequently will promote hepatic GSH release [4]. Although these *in vitro* studies clearly support corticosterone- and NORA-induced increased expression and secretion of IL-6 by B16-F10 cells, it is unclear if the same mechanisms are operating in the complexity of pathophysiological conditions in tumor-bearing mice. Nevertheless preliminary results obtained in our lab show that in B16 cells isolated from lung or liver metastatic foci (where metastatic cell GCR has been knocked down, previously to their inoculation, using specific shRNA), IL-6 expression and secretion is decreased (Estrela et al., unpublished results).

Additionally, potential cross-talk mechanisms, such as up-regulation of GCRs by IL-6 [54] or synergistic activation of IL-6 response element by IL-6 and glucocorticoids [55], could further potentiate this signaling mechanism. However, only corticosterone decreased tumor cell viability (Table 4) by activating mitochondria-dependent apoptosis (Figure 4 and Table 5). GSH (one of the key endogenous effectors involved in regulating activation of cell death pathways [56]), if maintained at high levels, was capable of preventing the corticosterone-induced apoptosis (Table 5). In particular mtGSH oxidation facilitates opening of the mitochondrial permeability transition pore complex and, in consequence, can be a causal factor in the mitochondrion-based mechanism that leads to cell death [3]. Thus corticosterone-induced increase in ROS generation must contribute to mtGSH depletion (Table 5). In this scenario several studies in thymocytes have implicated ROS in glucocorticoid-induced apoptosis signaling, and more recently it has been shown that hydrogen peroxide signaling is required for glucocorticoid-induced apoptosis in lymphoma cells [57]. Nevertheless the critical molecular targets or sensors of hydrogen peroxide during glucocorticoid-induced apoptosis signaling remain to be elucidated. It is noteworthy to mention that activation of neutral sphingomyelinase, which is induced by hydrogen peroxide, is required for glucocorticoid-induced apoptosis in thymocytes [57].

### IL-6, GSH, and the molecular mechanisms promoting tumor growth

Malignant melanoma cells, as well as practically all cancer cells, can release different growth factors and cytokines, which (in addition of their autocrine and paracrine effects) are potential systemic signals [58]. IL-6 serves as a major regulatory cytokine in the human body [59]. Solid tumor cells may secrete high levels of IL-6 (as shown here, a process stimulated by stress hormones, Table 1), which in turn promotes fundamental processes in cancer growth and metastasis including angiogenesis, proliferation, attachment, and invasion [13,49]. Previously we reported, in metastatic B16-F10 melanoma-bearing mice, that tumor IL-6 silencing causes a significant decrease in circulating IL-6 and in hepatic GSH efflux, and consequently an increase in liver GSH content [4]. Thus suggesting that tumor-derived IL-6 release is the main factor inducing GSH release form the liver. Nevertheless, it is plausible that in the liver, a major producer of IL-6, hepatic IL-6 may play a prevalent role particularly at early stages of metastatic invasion. A mechanism that may be further potentiated by tumor-derived factors [10,13]. Furthermore IL-6 may also provide tumor cells with mechanisms to escape cell death induced by stress and cytotoxic drugs, such as increased expression of several survival proteins, i.e. Bcl-2, Bcl-xL, Mcl-1, survivin, and XIAP [49]. The mechanism by which the transcription of specific eukaryotic genes is redox regulated is complex, however, it has been proposed that redox-sensitive transcription factors containing reactive thiols in their DNA binding regions (including e.g. NF-κB, AP-1, HIF-1, p53, or FoxO) play an essential role in this process [60]. Redox-sensitive cysteine residues sense and transduce changes in cellular redox status caused by the generation of ROS, reactive electrophilic species, reactive nitrogen species, and the presence of oxidized thiols [61]. Oxidation of such cysteines is converted into signals that control cell regulatory pathways and induction of gene expression [61]. Transcription factors including p53, NF-κB, and FoxO family can directly regulate the expression of different Bcl-2 family members [62]. Therefore it would be possible that GSH levels, by directly regulating the activity of redox-sensitive transcription factors and/or by decreasing ROS, may affect expression of proteins involved in regulating apoptosis. Furthermore, IL-6, as stressed in the introduction section, facilitates the interorgan transport of GSH to metastatic growing cells, thus favoring their growth and resistance [4]. In addition, as shown in the present report, metastatic cells with high GSH content are more resistant to corticosterone-induced apoptosis (Table 4).

### Physiological neuroendocrine systems and metastases growth

Within the tumor microenvironment ARs and GCRs in cancer, stromal cells, and tumor associated macrophages are activated by agonists from circulating blood; but, additionally, by catecholamines from sympathetic nerve fibers [40]. Additionally, it is also plausible that specific tissue/organ (such as liver or lung)-derived factors (still undefined) may contribute to GCR and AR expression by metastatic cells.

Moreover the brain can monitor immune status and sense peripheral cancer-related inflammation through two main pathways: neural and humoral. The neural mechanism would rely upon direct activation of vagus nerve afferent sensory fibers by cytokines, or indirectly through chemoreceptive cells located in vagal paraganglia [40]. Moreover neurotransmitters are known to regulate the migratory activity of tumor cells, and secondly, nerve fibers are used as routes for perineural invasion [63]. Therefore it is plausible that metastatic cell populations, as suggested by the present results, use physiological neuroendocrine mechanisms to promote growth of highly aggressive (high GSH content) cell subsets.

### Clinical applications

α- and β-ARs protein expression is associated with poor clinical outcome in breast cancer [64], thus suggesting a possible role for targeted therapy using ARs antagonists. In fact three recent population studies have translated laboratory investigations into a clinical setting and concur in presenting evidence that suggest a role for β-blockers in reducing metastases, tumor recurrence and specific mortality in breast cancer [65]. Besides RU-486, a GR antagonist, is used for treatment of several cancers, such as breast, ovarian, prostate, and glaucoma, and has been shown to sensitize renal carcinoma cells to TRAIL-induced apoptosis through up-regulation of DR5 and down-regulation of c-FLIP(L) and Bcl-2 [66]. Moreover, the first approved anti-IL-6 agent, tocilizumab, actually acts against the IL-6 receptor; whereas other anti-IL-6 compounds, including e.g. elsilimomab (a mouse monoclonal anti-IL-6 antibody) or CNTO 328 (an anti-IL-6 chimeric monoclonal antibody), are now following clinical trials (see http://clinicaltrials.gov). In addition to this we have reported a feasible strategy to deplete cytosolic and mitochondrial GSH in metastatic cells, under *in vivo* conditions, which includes a L-glutamine-enriched diet and an anti-Bcl-2 antisense therapy [3].

An obvious question is whether these effects are just present in the B16-F10 metastatic model. We have preliminary data in other experimental models, including non-metastatic human A375 melanoma and HT-29 colorectal cancer (xenografted into nu/nu nude mice) (tumor volumes >400 mm^3^) and metastatic Lewis lung carcinoma (10 days after i.v. inoculation into C57BL/6 mice, tail vein), where increased circulating levels of ACTH, corticosterone and NORA, associate with a decrease in hepatic GSH levels (in all cases as compared to non-tumor-bearing controls) (not shown). Thus suggesting that findings in the B16-F10 model are common to, at least, some other metastatic and non-metastatic models.

## Conclusions

**Figure 5:**
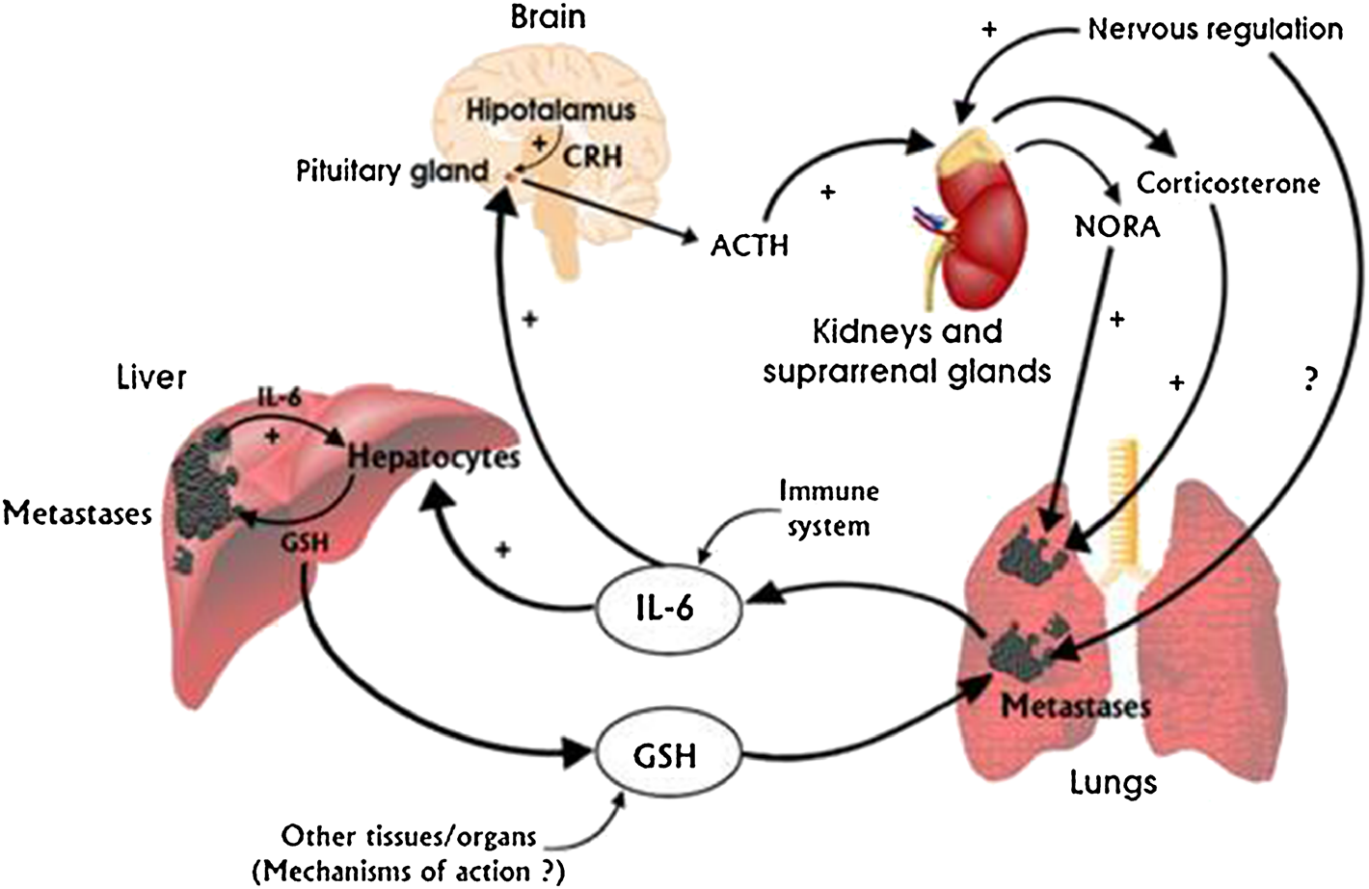
**The stress hormones/hypothalamic-pituitary-adrenal axis-dependent regulation of the IL-6/GSH interorgan cycling activity: a systemic mechanism promoting metastases growth.** IL-6 (mainly of tumor origin) potentiates the release of pituitary ACTH. Stress hormones released by the suprarenal glands upregulate IL-6 expression and secretion by metastatic cells, which in turns increases GSH release from the liver. Tumor GGT degrades plasma GSH, providing extra Cys for metastic cell GSH synthesis. Other (unknown) tumor-derived molecular signals, acting as GSH release activators in other cancers, the role of tissue specific microenvironments, or the possible influence of tumor innervation in metastatic cell behavior, are open questions.

The present results suggest the existence of an HPA-metastases circuit linked to the previously reported metastases-liver IL-6/GSH cycle. Figure 5 schematically summarizes these interorgan relationships.

## Abbreviations

IL: Interleukin; GSH: Glutathione; GGT: γ-glutamyl transpeptidase; B16-F10: B16 melanoma F10 subline; HPA: Hypothalamus-pituitary-adrenal axis; CRH: Corticotropin-releasing hormone; ACTH: Adrenocorticotropin hormone; NORA: Noradrenaline; RFP: Red fluorescence protein; PVN: Paraventricular nucleus; NF-κB: Nuclear factor-κB; AP-1: Activator protein-1; CREB: CAMP response element-binding protein; NF-IL-6: Nuclear factor for IL-6; GAPDH: Glyceraldehyde-3P-dehydrogenase; GCRs: Glucocorticoid receptors; ARs: Adrenoceptors; BSO: L-buthionine (SR)-sulphoximine; ROS: Reactive oxygen species; mtGSH: Mitochondrial GSH.

## Competing interests

The authors declare that they have no competing interests.

## Authors’ contributions

SLV, MLR, SM, JAP, MA, EO, and JME: Performed experiments. JME: Planned the studies and wrote the manuscript. All authors have read and approved the final manuscript.

## References

[B1] Meister A Selective modification of glutathione metabolism Science 1983 220 472 477 10.1126/science.6836290 6836290

[B2] Estrela JM Ortega A Obrador E Glutathione in cancer biology and therapy Crit Rev Clin Lab Sci 2006 43 143 181 10.1080/10408360500523878 16517421

[B3] Ortega A Mena S Estrela JM Glutathione in cancer cell death Cancers 2001 3 1285 1310 10.3390/cancers3011285 PMC3756414 24212662

[B4] Obrador E Benlloch M Pellicer JA Asensi M Estrela JM Intertissue flow of glutathione (GSH) as a tumor growth-promoting mechanism: interleukin 6 induces GSH release from hepatocytes in metastatic B16 melanoma-bearing mice J Biol Chem 2011 286 15716 15727 10.1074/jbc.M110.196261 21393247 PMC3091180

[B5] Zhang H Forman HJ Choi J Gamma-glutamyl transpeptidase in glutathione biosynthesis Methods Enzymol 2005 401 468 483 16399403 10.1016/S0076-6879(05)01028-1

[B6] Meister A Glutathione deficiency produced by inhibition of its synthesis, and its reversal; applications in research and therapy Pharmacol Ther 1991 51 155 194 10.1016/0163-7258(91)90076-X 1784629

[B7] Hanigan MH Expression of gamma-glutamyl transpeptidase provides tumor cells with a selective growth advantage at physiologic concentrations of cyst(e)ine Carcinogenesis 1995 16 181 185 10.1093/carcin/16.2.181 7859346

[B8] Obrador E Carretero J Ortega A Medina I Rodilla V Pellicer JA Estrela JM gamma-Glutamyl transpeptidase overexpression increases metastatic growth of B16 melanoma cells in the mouse liver Hepatology 2002 35 74 81 10.1053/jhep.2002.30277 11786961

[B9] Ballatori N Rebbeor JF Roles of MRP2 and oatp1 in hepatocellular export of reduced glutathione Semin Liver Dis 1998 18 377 387 10.1055/s-2007-1007171 9875555

[B10] Hodg DR Hurt EM Farrar WL The role of IL-6 and STAT3 in inflammation and cancer Eur J Cancer 2005 41 2502 2512 10.1016/j.ejca.2005.08.016 16199153

[B11] Barton BE Interleukin-6 and new strategies for the treatment of cancer, hyperproliferative diseases and paraneoplastic syndromes Expert Opin Ther Targets 2005 9 737 752 10.1517/14728222.9.4.737 16083340

[B12] Rose-John S Waetzig GH Scheller J Grötzinger J Seegert D The IL-6/sIL-6R complex as a novel target for therapeutic approaches Expert Opin Ther Targets 2007 11 613 624 10.1517/14728222.11.5.613 17465721

[B13] Ara T Declerck YA Interleukin-6 in bone metastasis and cancer progression Eur J Cancer 2010 46 1223 1231 10.1016/j.ejca.2010.02.026 20335016 PMC2917917

[B14] Emmenegger U Kerbel RS Cancer Chemotherapy counteracted Nature 2010 468 637 638 10.1038/468637a 21124441

[B15] Wang Y Niu XL Qu Y Wu J Zhu YQ Sun WJ Li LZ Autocrine production of interleukin-6 confers cisplatin and paclitaxel resistance in ovarian cancer cells Cancer Lett 2010 295 110 123 10.1016/j.canlet.2010.02.019 20236757

[B16] Sternberg EM Neural-immune interactions in health and disease J Clin Invest 1997 100 2641 2647 10.1172/JCI119807 9389725 PMC508465

[B17] Reiche EM Nunes SO Morimoto HK Stress, depression, the immune system, and cancer Lancet Oncol 2004 5 617 625 10.1016/S1470-2045(04)01597-9 15465465

[B18] Besedovsky HO Del Rey A Klusman I Furukawa H Monge Arditi G Kabiersch A Cytokines as modulators of the hypothalamus-pituitary-adrenal axis J Steroid Biochem Mol Biol 1991 40 613 618 10.1016/0960-0760(91)90284-C 1659887

[B19] Bethin KE Vogt SK Muglia LJ Interleukin-6 is an essential, corticotropin-releasing hormone-independent stimulator of the adrenal axis during immune system activation Proc Natl Acad Sci USA 2000 97 9317 9322 10.1073/pnas.97.16.9317 10922080 PMC16865

[B20] Fauci AS Mechanisms of the immunosuppressive and anti-inflammatory effects of glucocorticosteroids J Immunopharmacol 1978 1 1 25 401429 10.3109/08923977809027327

[B21] Herr I Pfitzenmaier J Glucocorticoid use in prostate cancer and other solid tumours: implications for effectiveness of cytotoxic treatment and metastases Lancet Oncol 2006 7 425 430 10.1016/S1470-2045(06)70694-5 16648047

[B22] Bernabé DG Tamae AC Biasoli ÉR Oliveira SH Stress hormones increase cell proliferation and regulates interleukin-6 secretion in human oral squamous cell carcinoma cells Brain Behav Immun 2011 25 574 583 10.1016/j.bbi.2010.12.012 21187140

[B23] Antoni MH Lutgendorf SK Cole SW Dhabhar FS Sephton SE McDonald PG Stefanek M Sood AK The influence of bio-behavioural factors on tumour biology: pathways and mechanisms Nat Rev Cancer 2006 6 240 248 10.1038/nrc1820 16498446 PMC3146042

[B24] Yang EV Kim SJ Donovan EL Chen M Gross AC Webster Marketon JI Barsky SH Glaser R Norepinephrine upregulates VEGF, IL-8, and IL-6 expression in human melanoma tumor cell lines: implications for stress-related enhancement of tumor progression Brain Behav Immun 2009 23 267 275 10.1016/j.bbi.2008.10.005 18996182 PMC2652747

[B25] Carretero J Obrador E Anasagasti MJ Martin JJ Vidal-Vanaclocha F Estrela JM Growth-associated changes in glutathione content correlate with liver metastatic activity of B16 melanoma cells Clin Exp Metastasis 1999 17 567 574 10.1023/A:1006725226078 10845555

[B26] Lachize S Apostolakis EM van der Laan S Tijssen AM Xu J de Kloet ER Meijer OC Steroid receptor coactivator-1 is necessary for regulation of corticotropin-releasing hormone by chronic stress and glucocorticoids Proc Natl Acad Sci USA 2009 106 8038 8042 10.1073/pnas.0812062106 19416907 PMC2683087

[B27] Veenema AH Reber SO Selch S Obermeier F Neumann ID Early life stress enhances the vulnerability to chronic psychosocial stress and experimental colitis in adult mice Endocrinology 2008 149 2727 2736 10.1210/en.2007-1469 18308845

[B28] New LS Chan EC Evaluation of BEH C18, BEH HILIC, and HSS T3 (C18) column chemistries for the UPLC-MS-MS analysis of glutathione, glutathione disulfide, and ophthalmic acid in mouse liver and human plasma Chromatogr Sci 2008 46 209 214 10.1093/chromsci/46.3.209 18334086

[B29] Asensi M Sastre J Pallardo FV Garcia De La Asuncion J Estrela JM Vina JA High-performance liquid chromatography method for measurement of oxidized glutathione in biological samples Anal Biochem 1994 217 323 328 10.1006/abio.1994.1126 8203763

[B30] Benlloch M Mena S Ferrer P Obrador E Asensi M Pellicer JA Carretero J Ortega A Estrela JM Bcl-2 and Mn-SOD antisense oligodeoxynucleotides and a glutamine-enriched diet facilitate elimination of highly resistant B16 melanoma cells by tumor necrosis factor-alpha and chemotherapy J Biol Chem 2006 281 69 79 16263711 10.1074/jbc.M507471200

[B31] Ortega AL Carretero J Obrador E Gambini J Asensi M Rodilla V Estrela JM Tumor cytotoxicity by endothelial cells. Impairment of the mitochondrial system for glutathione uptake in mouse B16 melanoma cells that survive after in vitro interaction with the hepatic sinusoidal endothelium J Biol Chem 2003 278 13888 13897 10.1074/jbc.M207140200 12578841

[B32] Sakakibara H Koyanagi A Suzuki T Suzuki A Ling L Shimoi K Effects of animal care procedures on plasma corticosterone levels in group-housed mice during the nocturnal active phase Exp Anim 2010 59 637 642 10.1538/expanim.59.637 21030792

[B33] Lucot JB Jackson N Bernatova I Morris M Measurement of plasma catecholamines in small samples from mice J Pharmacol Toxicol Methods 2005 52 274 277 10.1016/j.vascn.2004.11.004 16125626

[B34] Dobos J Kenessey I Tímár J Ladányi A Glucocorticoid receptor expression and antiproliferative effect of dexamethasone on human melanoma cells Pathol Oncol Res 2011 17 729 734 10.1007/s12253-011-9377-8 21455635

[B35] Ristic-Fira A Vujcic M Krstic-Demonacos M Kanazir D Identification and characterization of glucocorticoid receptors in B16 mouse melanoma cells Endocr Regul 1999 33 109 115 10571962

[B36] Tsuji M Kuno T Tanaka C Ichihashi M Mishima Y Beta-adrenergic receptors of B16 melanoma cell Arch Dermatol Res 1983 275 415 416 10.1007/BF00417345 6318672

[B37] Im A Appleman LJ Mifepristone: pharmacology and clinical impact in reproductive medicine, endocrinology and oncology Expert Opin Pharmacother 2010 11 481 488 10.1517/14656560903535880 20102310

[B38] Schuller HM Beta-adrenergic signaling, a novel target for cancer therapy? Oncotarget 2010 1 466 469 21317444 10.18632/oncotarget.182 PMC3248132

[B39] Smoak KA Cidlowski JA Mechanisms of glucocorticoid receptor signaling during inflammation Mech Aging Dev 2004 125 697 706 10.1016/j.mad.2004.06.010 15541765

[B40] Cole SW Sood AK Molecular pathways: beta-adrenergic signaling in cancer Clin Cancer Res 2012 18 1201 1206 10.1158/1078-0432.CCR-11-0641 22186256 PMC3294063

[B41] Boutillier AL Barthel F Roberts JL Loeffler JP Beta-adrenergic stimulation of cFOS via protein kinase A is mediated by cAMP regulatory element binding protein (CREB)-dependent and tissue-specific CREB-independent mechanisms in corticotrope cells J Biol Chem 1992 267 23520 23526 1331087

[B42] Matsusaka T Fujikawa K Nishio Y Mukaida N Matsushima K Kishimoto T Akira S Transcription factors NF-IL6 and NF-kappa B synergistically activate transcription of the inflammatory cytokines, interleukin 6 and interleukin 8 Proc Natl Acad Sci USA 1993 90 10193 10197 10.1073/pnas.90.21.10193 8234276 PMC47740

[B43] McEwen BS Physiology and neurobiology of stress and adaptation: central role of the brain Physiol Rev 2007 87 873 904 10.1152/physrev.00041.2006 17615391

[B44] Turnbull A Prehar S Kennedy A Little R Hopkins S Interleukin-6 is an afferent signal to the hypothalamo-pituitary-adrenal axis during local inflammation in mice Endocrinol 2003 144 1894 1906 10.1210/en.2002-220964 12697697

[B45] Lee JH Yoo SB Kim NY Cha MJ Jahng JW Interleukin-6 and the hypothalamic-pituitary-adrenal activation in a tumor bearing mouse Int J Neurosci 2008 118 355 364 10.1080/00207450701592915 18300009

[B46] Kageyama K Tamasawa N Suda T Signal transduction in the hypothalamic corticotropin-releasing factor system and its clinical implications Stress 2011 14 357 367 21438777 10.3109/10253890.2010.536279

[B47] Li YF He RR Tsoi B Li XD Li WX Abe K Kurihara H Anti-stress effects of carnosine on restraint-evoked immunocompromise in mice through spleen lymphocyte number maintenance PLoS One 2012 7 e33190 10.1371/journal.pone.0033190 22511917 PMC3325237

[B48] Sarabdjitsingh RA Kofink D Karst H de Kloet ER Joëls M Stress-induced enhancement of mouse amygdalar synaptic plasticity depends on glucocorticoid and ß-adrenergic activity PLoS One 2012 7 e42143 10.1371/journal.pone.0042143 22900007 PMC3416843

[B49] Moreno-Smith M Lutgendorf SK Sood AK Impact of stress on cancer metastasis Future Oncol 2010 6 1863 1881 10.2217/fon.10.142 21142861 PMC3037818

[B50] Tissing WJ Meijerink JP den Boer ML Pieters R Molecular determinants of glucocorticoid sensitivity and resistance in acute lymphoblastic leukemia Leukemia 2003 17 17 25 10.1038/sj.leu.2402733 12529655

[B51] Maung ZT Hogarth L Reid MM Proctor SJ Hamilton PJ Hall AG Raised intracellular glutathione levels correlate with in vitro resistance to cytotoxic drugs in leukaemic cells from patients with acute lymphoblastic leukemia Leukemia 1994 8 1487 1491 8090028

[B52] Anderer G Schrappe M Brechlin AM Lauten M Muti P Welte K Stanulla M Polymorphisms within glutathione S-transferase genes and initial response to glucocorticoids in childhood acute lymphoblastic leukaemia Pharmacogenetics 2000 10 715 726 10.1097/00008571-200011000-00006 11186134

[B53] Thaker PH Sood AK Neuroendocrine influences on cancer biology Semin Cancer Biol 2008 18 164 170 10.1016/j.semcancer.2007.12.005 18201896 PMC2424028

[B54] Snyers L De Wit L Content J Glucocorticoid up-regulation of high-affinity interleukin 6 receptors on human epithelial cells Proc Natl Acad Sci USA 1990 7 2838 2842 2157217 10.1073/pnas.87.7.2838 PMC53786

[B55] Takeda T Kurachi H Yamamoto T Nishio Y Nakatsuji Y Morishige K-I Miyake A Murata Y Crosstalk between the interleukin-6 (IL-6)-JAK-STAT and the glucocorticoid-nuclear receptor pathway: synergistic activation of IL-6 response element by IL-6 and glucocorticoid J Endocrinol 1998 159 323 330 10.1677/joe.0.1590323 9795374

[B56] Franco R Cidlowski JA Glutathione efflux and cell death Antioxid Redox Signal 2012 17 1676 1693 10.1089/ars.2011.4474 22656858 PMC3474185

[B57] Tome ME Jaramillo MC Briehl MM Hydrogen peroxide signaling is required for glucocorticoid-induced apoptosis in lymphoma cells Free Radic Biol Med 2011 51 2048 2059 10.1016/j.freeradbiomed.2011.09.002 21964507 PMC3208737

[B58] Lázár-Molnár E Hegyesi H Tóth S Falus A Autocrine and paracrine regulation by cytokines and growth factors in melanoma Cytokine 2000 12 547 554 10.1006/cyto.1999.0614 10843728

[B59] Sansone P Bromberg J Targeting the interleukin-6/Jak/stat pathway in human malignancies J Clin Oncol 2012 30 1005 1014 10.1200/JCO.2010.31.8907 22355058 PMC3341105

[B60] Arrigo AP Gene expression and the thiol redox state Free Radic Biol Med 1999 27 936 944 10.1016/S0891-5849(99)00175-6 10569626

[B61] Antelmann H Helmann JD Thiol-based redox switches and gene regulation Antioxid Redox Signal 2011 14 1049 1063 10.1089/ars.2010.3400 20626317 PMC3113447

[B62] Leibowitz B Yu J Mitochondrial signaling in cell death via the Bcl-2 family Cancer Biol Ther 2010 9 417 422 10.4161/cbt.9.6.11392 20190564 PMC2874116

[B63] Mancino M Ametller E Gascón P Almendro V The neuronal influence on tumor progression Biochim Biophys Acta 1816 2011 105 118 10.1016/j.bbcan.2011.04.005 21616127

[B64] Powe DG Voss MJ Habashy HO Zänker KS Green AR Ellis IO Entschladen F Alpha- and beta-adrenergic receptor (AR) protein expression is associated with poor clinical outcome in breast cancer: an immunohistochemical study Breast Cancer Res Treat 2011 130 457 463 10.1007/s10549-011-1371-z 21298476

[B65] Powe DG Entschladen F Targeted therapies: Using β-blockers to inhibit breast cancer progression Nat Rev Clin Oncol 2011 8 511 512 10.1038/nrclinonc.2011.123 21808268

[B66] Min KJ Jang J Lee JT Choi KS Kwon TK Glucocorticoid receptor antagonist sensitizes TRAIL-induced apoptosis in renal carcinoma cells through up-regulation of DR5 and down-regulation of c-FLIP(L) and Bcl-2 J Mol Med (Berl) 2012 90 309 319 10.1007/s00109-011-0821-8 22008998

